# Supplementary figures and images for: Skewed perception of personal behaviour as a contributor to antibiotic resistance and underestimation of the risks
Source: PLoS One. 2023 Nov 2;18(11):e0293186. doi: 10.1371/journal.pone.0293186 (PMC10621963; doi:10.1371/journal.pone.0293186)

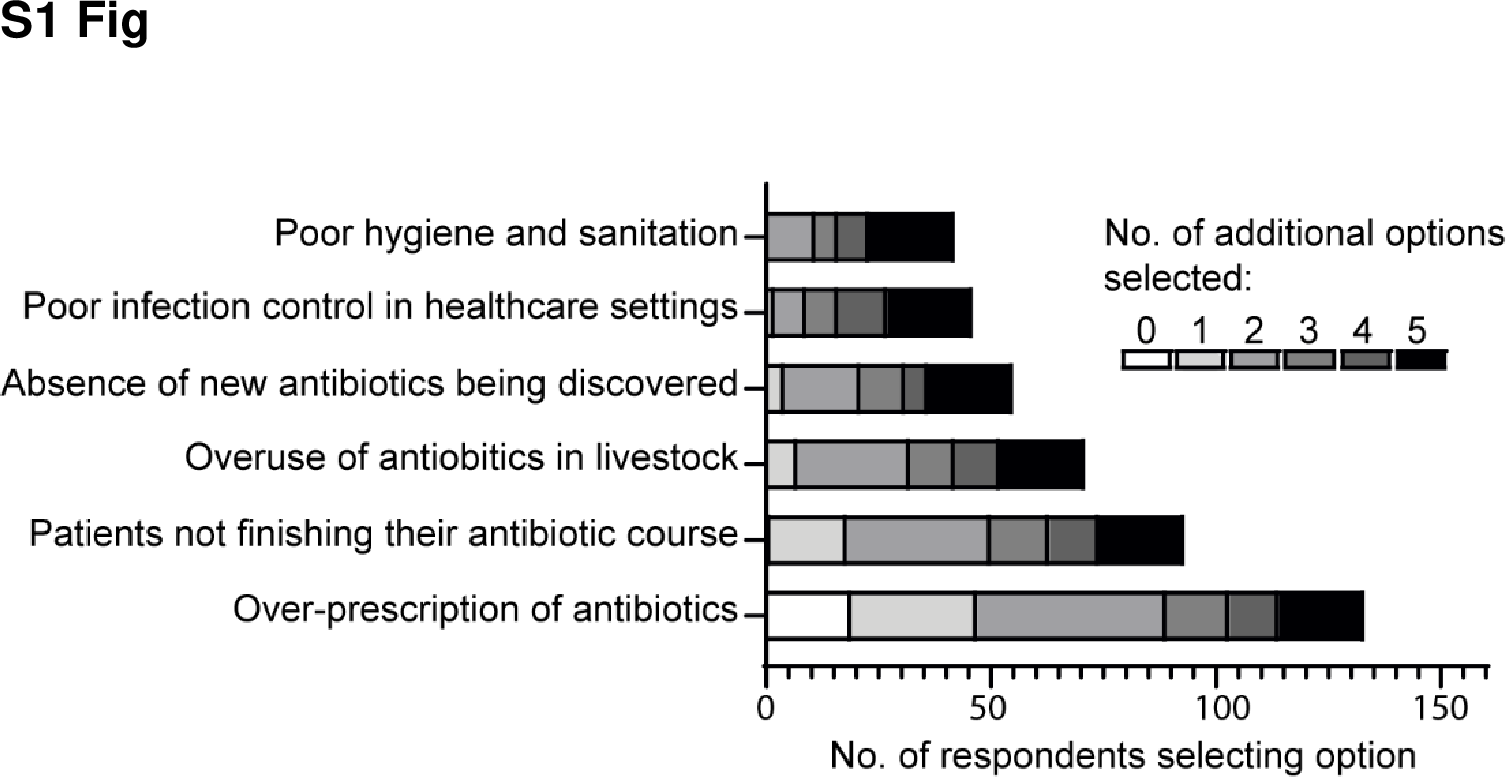

Supplement: S1 Fig — Participants who reported that they had previously received antibiotics in the past (n = 141) were asked ‘Which of the following factors do you think would impact antibiotic resistance (tick all that apply)?’. The number of respondents selecting each option is shown, along with an indication of how many additional options were chosen in parallel. Two individuals selected the option ‘none of the above’ (not shown). The remaining 162 respondents selected one or more of the 6 options as shown. Two-way ANOVA p<0.0009 for ‘number of additional options’ or each of the contributors as a factor. Significant differences (Tukey’s post-hoc test) were observed between the number of additional options selected by those choosing ‘over-prescription of antibiotics’ compared with those selecting societal factors such as ‘poor hygiene and sanitation’ (p = 0.0016), ‘poor infection control in healthcare settings’ (p = 0.0027) and ‘absence of new antibiotics being discovered’ (p = 0.008). (TIF) [file pone.0293186.s001.tif]

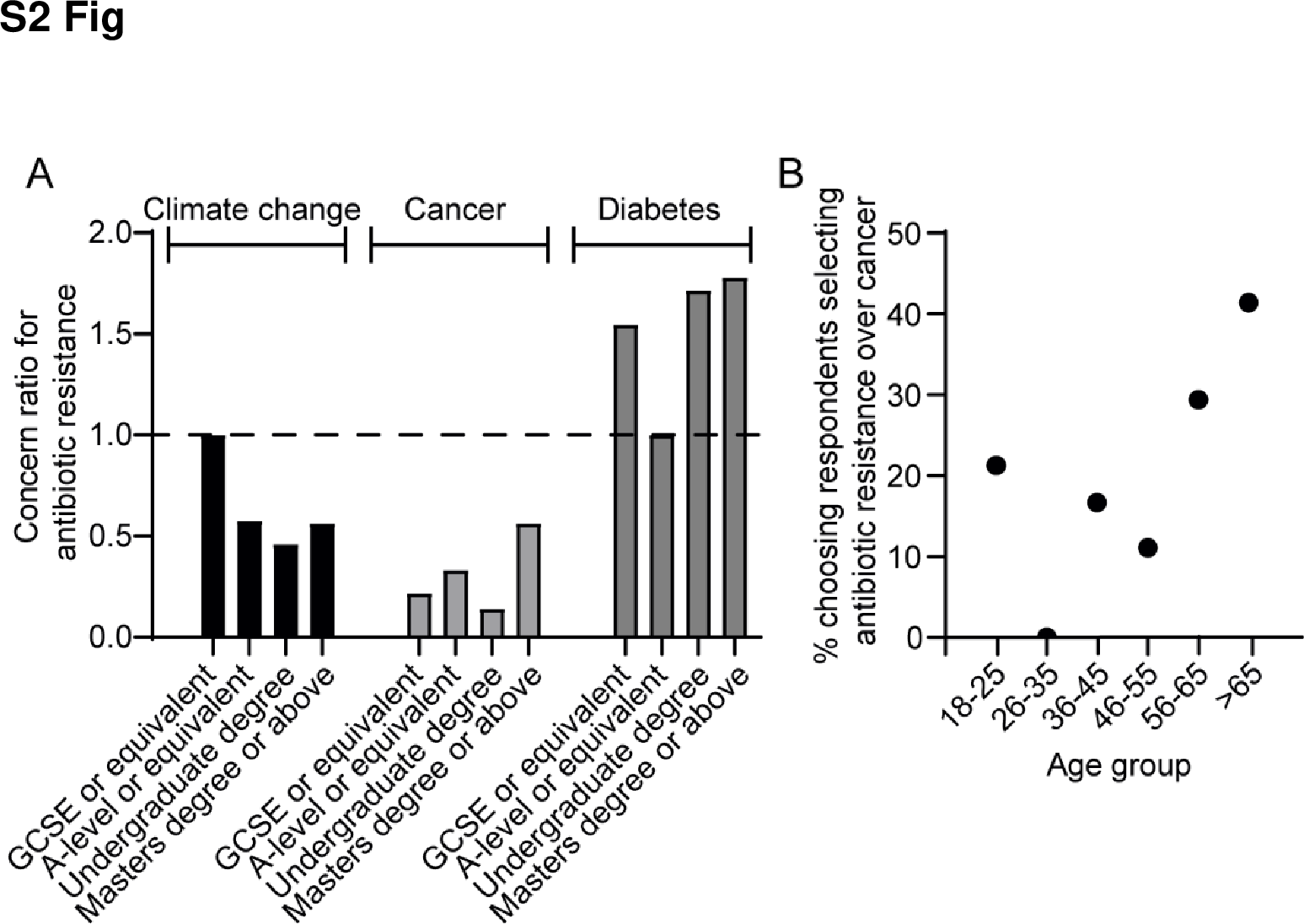

Supplement: S2 Fig — (A) CRAR values (as Fig 1) were calculated for AR compared with climate change, cancer and diabetes and responses compared across different educational backgrounds as indicated. Only 2 respondents had an educational background of ‘none’; both were more concerned with AR compared with climate change and diabetes but considered cancer more concerning than AR; due to the small number of respondents in these groups, these data were omitted from the graph. Values greater than 1 indicate a bias towards concern about AR compared with the alternative factor. (B) % of respondents in each age group selecting AR as greater concern than cancer. See Table 1 for numbers of individuals in each category; all respondents provided an answer to this question. Pearson’s two-tailed correlation p = 0.14. (TIF) [file pone.0293186.s002.tif]
