# Supplementary material for: Skewed perception of personal behaviour as a contributor to antibiotic resistance and underestimation of the risks
Source: PLoS One. 2023 Nov 2;18(11):e0293186. doi: 10.1371/journal.pone.0293186 (PMC10621963; doi:10.1371/journal.pone.0293186)
Supplement: S1 Table — 1Y = Yes, N = No. Numbers indicate number of respondents providing this answer. Numbers in parentheses indicate percentage of group providing this answer. 2Fisher’s exact test, 3Chi-squared test. 4Comparing age groups 18–45 with those aged 46 and over. 5Comparing groups with and without a minimum degree level qualification. NS p>0.05, *p<0.05, **p<0.01. (DOCX) [file pone.0293186.s003.docx]

**Supplementary Table 1. Responses to questions relating to personal use of antibiotics**

| Q1. Have you ever taken a course of antibiotics?  Q2. How often have you taken antibiotics in the past 5 years?  Q3. Did you get advice on how to take the antibiotics?  Q4. If you felt better halfway through the course would you stop taking the antibiotics?  Q5. Which of the following do you think impact antibiotic resistance (select all that apply)? | | | | | | | | | | | | | | | | | | | |
| --- | --- | --- | --- | --- | --- | --- | --- | --- | --- | --- | --- | --- | --- | --- | --- | --- | --- | --- | --- |
|  | Q1 | | Q2 | Q3 | | Q4. | | Q5. | | | | | | | | | | | |
|  |  | |  |  | |  | | Over-prescription of antibiotics | | Patients not finishing course | | Overuse of Antibiotics in livestock | | Absence of antibiotics being discovered | | Poor infection control in healthcare settings | | Poor hygiene and sanitation | |
| Male | Y^1^  50  (79) | N^1^  13  (21) | 0: 24  1-5: 38  >5: 1 | Y  38  (97) | N  1  (3) | Y  9  (14) | N  54  (86) | Y  58  (92) | N  5  (8) | Y  34  (54) | N  29  (46) | Y  32  (51) | N  31  (49) | Y  27  (43) | N  36  (57) | Y  22  (35) | N  41  (65) | Y  19  (30) | N  44  (70) |
| Female | Y  91  (90) | N  10  (10) | 0: 23  1-5: 70  >5: 8 | Y  66  (85) | N  12  (15) | Y  6  (6) | N  95  (94) | Y  94  (93) | N  7  (7) | Y  73  (72) | N  28  (18) | Y  50  (50) | N  51  (50) | Y  33  (33) | N  68  (67) | Y  35  (35) | N  66  (65) | Y  33  (33) | N  68  (66) |
| M/F different? | NS^2^ | | *^3^ | NS^2^ | | NS^2^ | | NS^2^ | | *^2^ | | NS^2^ | | NS^2^ | | NS^2^ | | NS^2^ | |
| 18-25 | Y  33  (70) | N  14  (30) | 0: 16  1-5: 28  >5: 3 | Y  27  (82) | N  5  (18) | Y  7  (15) | N  40  (85) | Y  43  (91) | N  4  (8) | Y  41  (87) | N  6  (13) | Y  18  (38) | N  29  (62) | Y  16  (34) | N  31  (66) | Y  19  (40) | N  28  (60) | Y  19  (40) | N  28  (60) |
| 26-35 | Y  17  (85) | N  3  (15) | 0: 4  1-5: 16  >5: 0 | Y  13  (81) | N  3  (19) | Y  2  (10) | N  18  (90) | Y  17  (85) | N  3  (15) | Y  11  (55) | N  9  (45) | Y  8  (40) | N  12  (60) | Y  7  (35) | N  13  (65) | Y  8  (40) | N  12  (60) | Y  7  (35) | N  13  (65) |
| 36-45 | Y  22  (92) | N  2  (8) | 0: 4  1-5: 19  >5: 1 | Y  19  (95) | N  1  (5) | Y  1  (4) | N  23  (96) | Y  23  (96) | N  1  (4) | Y  15  (63) | N  9  (37) | Y  13  (54) | N  11  (46) | Y  6  (25) | N  18  (75) | Y  5  (21) | N  19  (79) | Y  4  (17) | N  20  (83) |
| 46-55 | Y  25  (93) | N  2  (7) | 0: 11  1-5: 15  >5: 1 | Y  15  (94) | N  1  (6) | Y  3  (11) | N  24  (89) | Y  27  (100) | N  0  (0) | Y  14  (52) | N  13  (48) | Y  14  (52) | N  13  (48) | Y  8  (30) | N  19  (70) | Y  12  (44) | N  15  (56) | Y  9  (33) | N  18  (67) |
| 56-65 | Y  17  (100) | N  0  (0) | 0: 3  1-5: 13  >5: 1 | Y  11  (79) | N  3  (21) | Y  1  (6) | N  16  (94) | Y  15  (88) | N  2  (12) | Y  10  (59) | N  7  (41) | Y  10  (59) | N  7  (41) | Y  9  (53) | N  8  (47) | Y  7  (42) | N  10  (58) | Y  6  (35) | N  11  (65) |
| >65 | Y  27  (93) | N  2  (7) | 0: 9  1-5: 17  >5: 3 | Y  19  (100) | N  0  (0) | Y  1  (3) | N  28  (97) | Y  27  (93) | N  2  (7) | Y  16  (55) | N  13  (45) | Y  19  (66) | N  10  (34) | Y  14  (48) | N  15  (52) | Y  6  (21) | N  23  (79) | Y  7  (24) | N  22  (76) |
| Age factor?^4^ | **^2^ | | NS^3^ | NS^2^ | | NS^2^ | | NS^2^ | | *^2^ | | NS^2^ | | NS^2^ | | NS^2^ | | NS^2^ | |
|  | Q1 | | Q2 | Q3 | | Q4. | | Q5. | | | | | | | | | | | |
|  |  | |  |  | |  | | Over-prescription of antibiotics | | Patients not finishing course | | Overuse of Antibiotics in livestock | | Absence of antibiotics being discovered | | Poor infection control in healthcare settings | | Poor hygiene and sanitation | |
| No education | Y  2  (100) | N  0  (0) | 0: 0  1-5: 2  >5: 0 | Y  2  (100) | N  0  (0) | Y  0  (0) | N  2  (100) | Y  2  (100) | N  0  (0) | Y  0  (0) | N  2  (100) | Y  0  (0) | N  2  (100) | Y  0  (0) | N  2  (100) | Y  0  (0) | N  2  (100) | Y  0  (0) | N  2  (100) |
| GCSE or equivalent | Y  20  (71) | N  8  (29) | 0: 10  1-5: 15  >5: 3 | Y  18  (100) | N  0  (0) | Y  5  (18) | N  23  (78) | Y  24  (86) | N  4  (14) | Y  16  (57) | N  12  (43) | Y  10  (36) | N  18  (64) | Y  8  (29) | N  20  (71) | Y  9  (32) | N  19  (68) | Y  8  (29) | N  20  (71) |
| A-level or equivalent | Y  45  (90) | N  7  (10) | 0: 13  1-5: 35  >5: 4 | Y  33  (87) | N  5  (13) | Y  6  (12) | N  46  (88) | Y  48  (92) | N  4  (8) | Y  32  (62) | N  20  (38) | Y  26  (50) | N  26  (50) | Y  16  (31) | N  36  (69) | Y  20  (38) | N  32  (62) | Y  15  (29) | N  37  (71) |
| Undergraduate degree | Y  52  (91) | N  5  (9) | 0: 17  1-5: 39  >5: 1 | Y  38  (93) | N  3  (7) | Y  1  (2) | N  56  (98) | Y  55  (96) | N  2  (4) | Y  43  (75) | N  14  (25) | Y  30  (53) | N  27  (47) | Y  28  (49) | N  29  (51) | Y  23  (40) | N  34  (60) | Y  25  (44) | N  32  (66) |
| Master’s degree or above | Y  22  (88) | N  3  (12) | 0: 7  1-5: 17  >5: 1 | Y  13  (72) | N  5  (28) | Y  3  (12) | N  22  (88) | Y  23  (92) | N  2  (8) | Y  16  (64) | N  9  (36) | Y  16  (64) | N  9  (36) | Y  8  (32) | N  17  (68) | Y  5  (20) | N  20  (80) | Y  4  (16) | N  21  (84) |
| Education factor? ^5^ | NS^2^ | | NS^3^ | NS^2^ | | NS^2^ | | NS^2^ | | NS^2^ | | NS^2^ | | NS^2^ | | NS^2^ | | NS^2^ | |
