# Supplementary material for: Skewed perception of personal behaviour as a contributor to antibiotic resistance and underestimation of the risks
Source: PLoS One. 2023 Nov 2;18(11):e0293186. doi: 10.1371/journal.pone.0293186 (PMC10621963; doi:10.1371/journal.pone.0293186)
Supplement: S2 Table — 1Numbers indicate number of respondents providing this answer. Numbers in parentheses indicate percentage of group providing this answer. 2Full text for this question was “What do you estimate will be the number of deaths worldwide attributed to antibiotic resistance by 2050, according to Public Health England? Answer to the nearest million.”Group mean is shown with range in parentheses. 3Chi-squared test, 4Mann-Whitney U-test, 5Fisher’s exact test. 6Comparing age groups 18–45 with those aged 46 and over. 7Comparing groups with and without a minimum degree level qualification. NS p>0.05, *p<0.05, ***p<0.005. (DOCX) [file pone.0293186.s004.docx]

**Supplementary Table 2. Responses to questions relating to AR risk perception**

|  | What do you estimate is the current number of deaths worldwide attributed to antibiotic resistance?^1^ | Estimated deaths worldwide by 2050^2^ | Which of the following are you more concerned about?^1^ | | | | | | Which of the following are you more concerned about? Amount of antibiotics prescribed to humans / amount of antibiotics prescribed to animals^1^ |
| --- | --- | --- | --- | --- | --- | --- | --- | --- | --- |
|  |  |  | AR | Climate change | AR | Cancer | AR | Diabetes |  |
| Male | Under 250,000: 8 (16)  250,000–500,000: 16 (25)  500,001–1,000,000: 20 (32)  Over 1,000,000: 19 (30) | 7,406,250  (750,000 – 50,000,000) | 17 (27) | 46 (73) | 19 (30) | 44 (70) | 38 (60) | 25 (40) | 41 (65)/ 22 (35) |
| Female | Under 250,000: 26 (26)  250,000–500,000: 35 (35)  500,001–1,000,000: 28 (28)  Over 1,000,000: 12 (12) | 5,826,702  (10,000 - 50,000,000) | 45 (45) | 56 (55) | 15 (15) | 86 (85) | 59 (58) | 42 (42) | 67 (66)/ 34 (34) |
| M/F different? | *^3^ | ***^4^ | *^5^ | | *^5^ | | NS^5^ | | NS^5^ |
| 18-25 | Under 250,000: 2 (4)  250,000–500,000: 24 (51)  500,001–1,000,000: 16 (34)  Over 1,000,000: 5 (11) | 7,938,889  (500,000 – 50,000,000) | 16 (34) | 31 (66) | 10 (21) | 37 (79) | 31 (66) | 16 (34) | 35 (74)/ 12 (26) |
| 26-35 | Under 250,000: 10 (50)  250,000–500,000: 4 (20  500,001–1,000,000: 5 (25)  Over 1,000,000: 1 (5) | 9,411,765  (1,000,000 – 50,000,000) | 7 (35) | 13 (65) | 0 (0) | 20 (100) | 7 (35) | 13 (65) | 13 (65)/ 7 (35) |
| 36-45 | Under 250,000: 10 (42)  250,000–500,000: 4 (17)  500,001–1,000,000: 5 (21)  Over 1,000,000: 5 (21) | 3,434,783  (500,000 – 10,000,000) | 8 (33) | 16 (67) | 4 (17) | 20 (83) | 14 (58) | 10 (42) | 13 (54)/ 11 (46) |
| 46-55 | Under 250,000: 6 (22)  250,000–500,000: 7 (26)  500,001–1,000,000: 8 (30)  Over 1,000,000: 6 (22) | 4,088,400  (10,000 – 20,000,000) | 12 (44) | 15 (66) | 3 (11) | 24 (89) | 16 (59) | 11 (41) | 15 (56)/ 12 (44) |
| 56-65 | Under 250,000: 2 (12)  250,000–500,000: 5 (29)  500,001–1,000,000: 5 (29)  Over 1,000,000: 5 (29) | 7,615,385  (1,000,000 – 50,000,000) | 7 (41) | 10 (59) | 5 (29) | 12 (71) | 10 (59) | 7 (41) | 14 (82)/ 3 (18) |
| >65 | Under 250,000: 4 (14)  250,000–500,000: 7 (24)  500,001–1,000,000: 9 (31)  Over 1,000,000: 9 (31) | 6,111,111  (1,000,000 – 20,000,000) | 12 (41) | 17 (59) | 12 (41) | 17 (59) | 19 (66) | 10 (34) | 18 (62)/ 11 (38) |
| Age factor?^6^ | NS^3^ | NS^4^ | NS^5^ | | NS^5^ | | NS^5^ | | NS^5^ |
| No education | Under 250,000: 0 (0)  250,000–500,000: 1 (50)  500,001–1,000,000: 1 (50)  Over 1,000,000: 0 (0) | 4,500,000  (4,000,000 – 5,000,000) | 2 (100) | 0 (0) | 0 (0) | 2 (100) | 2 (100) | 0 (0) | 2 (100)/ 0 (0) |
| GCSE or equivalent | Under 250,000: 5 (18)  250,000–500,000: 5 (18)  500,001–1,000,000: 12 (43)  Over 1,000,000: 6 (21) | 5,150,357  (10,000 – 20,000,000) | 14 (50) | 14 (50) | 5 (18) | 23 (82) | 17 (61) | 11 (39) | 19 (68)/ 9 (32) |
| A-level or equivalent | Under 250,000: 5 (10)  250,000–500,000: 18 (35)  500,001–1,000,000: 14 (27)  Over 1,000,000: 15 (29) | 6,750,000  (500,000 – 50,000,000) | 19 (37) | 33 (63) | 13 (25) | 39 (75) | 26 (50) | 26 (50) | 40 (77)/ 12 (33) |
| Undergraduate degree | Under 250,000: 16 (28)  250,000–500,000: 19 (33)  500,001–1,000,000: 13 (23)  Over 1,000,000: 9 (16) | 5,443,396 (500,000 – 50,000,000) | 18 (33) | 39 (67) | 7 (12) | 50 (88) | 36 (63) | 21 (37) | 35 (61)/ 22 (39) |
| Master’s degree or above | Under 250,000: 8 (32)  250,000–500,000: 8 (32)  500,001–1,000,000: 8 (32)  Over 1,000,000: 1 (4) | 9,863,636 (1,000,000 – 50,000,000) | 9 (36) | 16 (64) | 9 (36) | 16 (64) | 16 (64) | 9 (36) | 12 (48)/ 13 (52) |
| Education factor?^7^ | *^3^ | NS^4^ | NS^5^ | | *^5^ | | NS^5^ | | *^5^ |
