# Supplementary material for: Skewed perception of personal behaviour as a contributor to antibiotic resistance and underestimation of the risks
Source: PLoS One. 2023 Nov 2;18(11):e0293186. doi: 10.1371/journal.pone.0293186 (PMC10621963; doi:10.1371/journal.pone.0293186)
Supplement: S1 Appendix — (DOCX) [file pone.0293186.s005.docx]

**S1 Appendix. Questions included in the questionnaire.**

1. What gender do you identify as?

- Male
- Female
- Transgender
- Other

2. How old are you?

- 18-25
- 26-35
- 36-45
- 46-55
- 56-65
- Over 65

3. What is the highest level of formal education you have attained?

- GCSE or equivalent
- A-level or equivalent
- Undergraduate degree
- Master’s degree or higher

4. Which County do you live in?

5. Have you ever taken a course of antibiotics?

Yes/No

6. If yes, when was the last time you took antibiotics?

7. How often have you taken antibiotics in the past 5 years?

- Never
- 1-5 prescriptions
- Over 5 prescriptions

8. Did you get advice about how to take the antibiotics?

Yes/No

9. If yes, from where did you receive this advice?

- Doctor
- Internet
- Leaflet
- Friend/family

10. What are you personally most likely to take antibiotics for (e.g. colds, coughs, infections, etc.)?

11. When prescribed a course of antibiotics, if you felt better halfway through the course, would you stop taking the antibiotics?

Yes/No

12. If yes, what would you do with the left-over medication?

- Return them to the pharmacy
- Throw them away
- Keep them
- Give them to a friend who has a similar illness

13. Do you think it is acceptable to take antibiotics that were given to a friend or family member, as long as they were used to treat the same illness?

Yes/No

14. How much knowledge would you say you have about antibiotic resistance?

- None
- Some
- Lots

15. Which of the following factors do you think would impact antibiotic resistance (tick all that apply)?

- Over-prescription of antibiotics
- Patients not finishing the entire antibiotic course
- Overuse of antibiotics in livestock and fish farming
- Poor infection control in healthcare settings
- Poor hygiene and sanitation
- Absence of new antibiotics being discovered
- None of the above

16. What do you estimate is the current number of deaths worldwide attributed to antibiotic resistance?

- Under 250,000
- 250,000 to 500,000
- 500,001 to 1,000,00
- Over 1,000,000

17. What do you estimate will be the number of deaths worldwide attributed to antibiotic resistance by 2050, according to Public Health England? Answer to the nearest million.

18. Which of the following two options are you more concerned about?

- Antibiotic resistance
- Climate change

19. Which of the following two options are you more concerned about?

- Antibiotic resistance
- Cancer

20. Which of the following two options are you more concerned about?

- Antibiotic resistance
- Diabetes

21. Which of the following two options are you more concerned about?

- The amount of antibiotics being prescribed to humans
- The amount of antibiotics being prescribed to animals
