# Supplementary material for: Skewed perception of personal behaviour as a contributor to antibiotic resistance and underestimation of the risks
Source: PLoS One. 2023 Nov 2;18(11):e0293186. doi: 10.1371/journal.pone.0293186 (PMC10621963; doi:10.1371/journal.pone.0293186)
Supplement: Attachment — Submitted filename: Response to Reviewers.docx [file pone.0293186.s006.docx]

**Response to Reviewers**

**Journal requirements**

1. Please ensure that your manuscript meets PLOS ONE's style requirements, including those for file naming. The PLOS ONE style templates can be found at

https://journals.plos.org/plosone/s/file?id=wjVg/PLOSOne_formatting_sample_main_body.pdf and

<https://journals.plos.org/plosone/s/file?id=ba62/PLOSOne_formatting_sample_title_authors_affiliations.pdf>

*Having re-checked the style requirements since submission, we have made a minor amendment to correct ‘Materials and Methods’ to ‘Materials and methods’ (line 88) to conform to sentence case. In addition, the corresponding authors initials have been added in parentheses after the email address (line 14). The remainder of the manuscript conforms to PLOS ONE’s style requirements.*

2. We note that you have stated that you will provide repository information for your data at acceptance. Should your manuscript be accepted for publication, we will hold it until you provide the relevant accession numbers or DOIs necessary to access your data. If you wish to make changes to your Data Availability statement, please describe these changes in your cover letter and we will update your Data Availability statement to reflect the information you provide.

*We confirm that the data are available via access through the following DOI:* [*https://doi.org/10.18746/bmth.data.00000316*](https://eur02.safelinks.protection.outlook.com/?url=https%3A%2F%2Fdoi.org%2F10.18746%2Fbmth.data.00000316&data=05%7C01%7Csbuchan%40bournemouth.ac.uk%7Cbf4344f6fc8e49c4bc8508db9a4d3358%7Cede29655d09742e4bbb5f38d427fbfb8%7C0%7C0%7C638273428578257406%7CUnknown%7CTWFpbGZsb3d8eyJWIjoiMC4wLjAwMDAiLCJQIjoiV2luMzIiLCJBTiI6Ik1haWwiLCJXVCI6Mn0%3D%7C3000%7C%7C%7C&sdata=teFZ8ZBLi8Gs%2FriZCWs3tcwcNIfZUpTlYHGMV889buw%3D&reserved=0)*. We would be grateful if the Data Availability statement could be updated to reflect this as follows ‘Raw data are available through Bournemouth University’s BORDaR database at* [*https://doi.org/10.18746/bmth.data.00000316*](https://eur02.safelinks.protection.outlook.com/?url=https%3A%2F%2Fdoi.org%2F10.18746%2Fbmth.data.00000316&data=05%7C01%7Csbuchan%40bournemouth.ac.uk%7Cbf4344f6fc8e49c4bc8508db9a4d3358%7Cede29655d09742e4bbb5f38d427fbfb8%7C0%7C0%7C638273428578257406%7CUnknown%7CTWFpbGZsb3d8eyJWIjoiMC4wLjAwMDAiLCJQIjoiV2luMzIiLCJBTiI6Ik1haWwiLCJXVCI6Mn0%3D%7C3000%7C%7C%7C&sdata=teFZ8ZBLi8Gs%2FriZCWs3tcwcNIfZUpTlYHGMV889buw%3D&reserved=0)*’.*

3. We note that you have included the phrase “data not shown” in your manuscript. Unfortunately, this does not meet our data sharing requirements. PLOS does not permit references to inaccessible data. We require that authors provide all relevant data within the paper, Supporting Information files, or in an acceptable, public repository. Please add a citation to support this phrase or upload the data that corresponds with these findings to a stable repository (such as Figshare or Dryad) and provide and URLs, DOIs, or accession numbers that may be used to access these data. Or, if the data are not a core part of the research being presented in your study, we ask that you remove the phrase that refers to these data.

*We apologise for this oversight. The term ‘data not shown’ appeared three times in the submitted manuscript. In response, the first and second uses of the phrase (lines 135 and 216 in the original document, corresponding to lines 137 and 221 in the tracked version), have been removed since in each case the pertinent data are included within the sentence itself. The final ‘data not shown’ phrase has also been removed (line 327 in the original document and line 332 in the tracked version) as the statement is adequately supported by S2 Table which is already referred to in the same sentence.*

4. Please review your reference list to ensure that it is complete and correct. If you have cited papers that have been retracted, please include the rationale for doing so in the manuscript text, or remove these references and replace them with relevant current references. Any changes to the reference list should be mentioned in the rebuttal letter that accompanies your revised manuscript. If you need to cite a retracted article, indicate the article’s retracted status in the References list and also include a citation and full reference for the retraction notice.

*In response to this comment, we have re-checked all references. As a result, some extraneous details have been removed including PMCID and PMID data and Epub dates. In addition, references 9, 16, 20, 26-28, 30 and 33 have been amended to sentence case. In response to a comment from the reviewers (see below), we realise a citation was missing in line 421 of the original manuscript (line 434 in the tracked version). This citation has been added as new citation 43. Subsequent citation numbering has been amended both in the text of the manuscript and in the references list. The rationale for addition of this reference is explained in the response to the reviewer below. All references are still cited in numerical order. References 1, 11, 27, 36, 38 and 42-45 have been amended and additional information added to comply with formatting requirements for published media/on-line articles. All these changes are tracked within the revised manuscript.*

**Additional Editor Comments:**

Please respond to the reviewers decision. *Please see responses in italics below.*

[Note: HTML markup is below. Please do not edit.]

**Reviewers' comments:**

Reviewer's Responses to Questions

**Comments to the Author**

1. Is the manuscript technically sound, and do the data support the conclusions?

The manuscript must describe a technically sound piece of scientific research with data that supports the conclusions. Experiments must have been conducted rigorously, with appropriate controls, replication, and sample sizes. The conclusions must be drawn appropriately based on the data presented.

Reviewer #1: Yes

Reviewer #2: Yes

2. Has the statistical analysis been performed appropriately and rigorously?

Reviewer #1: Yes

Reviewer #2: I Don't Know

3. Have the authors made all data underlying the findings in their manuscript fully available?

The [PLOS Data policy](https://eur02.safelinks.protection.outlook.com/?url=http%3A%2F%2Fwww.plosone.org%2Fstatic%2Fpolicies.action%23sharing&data=05%7C01%7Csbuchan%40bournemouth.ac.uk%7C7a739822b41c4ef556d408dba8d5a3b5%7Cede29655d09742e4bbb5f38d427fbfb8%7C0%7C0%7C638289408019929467%7CUnknown%7CTWFpbGZsb3d8eyJWIjoiMC4wLjAwMDAiLCJQIjoiV2luMzIiLCJBTiI6Ik1haWwiLCJXVCI6Mn0%3D%7C3000%7C%7C%7C&sdata=9IpUmmEqntaTj0RFbEtJehVAzt%2FB%2FazIzb6SjuVwtF0%3D&reserved=0) requires authors to make all data underlying the findings described in their manuscript fully available without restriction, with rare exception (please refer to the Data Availability Statement in the manuscript PDF file). The data should be provided as part of the manuscript or its supporting information, or deposited to a public repository. For example, in addition to summary statistics, the data points behind means, medians and variance measures should be available. If there are restrictions on publicly sharing data—e.g. participant privacy or use of data from a third party—those must be specified.

Reviewer #1: Yes

Reviewer #2: Yes

4. Is the manuscript presented in an intelligible fashion and written in standard English?

PLOS ONE does not copyedit accepted manuscripts, so the language in submitted articles must be clear, correct, and unambiguous. Any typographical or grammatical errors should be corrected at revision, so please note any specific errors here.

Reviewer #1: Yes

Reviewer #2: Yes

*We thank the reviewers for their positive responses to questions 1-4. No further comment is required from the authors to these questions above.*

5. Review Comments to the Author

Please use the space provided to explain your answers to the questions above. You may also include additional comments for the author, including concerns about dual publication, research ethics, or publication ethics. (Please upload your review as an attachment if it exceeds 20,000 characters).

*Our comments are provided in italics below. This information is also supplied in a separate file ‘Response to Reviewers’.*

Reviewer #1: Good study. The proposal gives incite into the practices in the mentioned geographic area and lays stress on the points which can be acted upon. This will further strengthen the fight against antimicrobial resistance.

Reviewer #2: This is a study of the public’s knowledge, attitudes and habits towards antibiotic use and their perceptions of antibiotic resistance, including personal and societal factors that might affect resistance development. The study also assessed the participants’ perception of the risk of AR relative to other crises such as climate change and cancer, in addition to the absolute risk of AR in terms of the number of deaths now and in the future.

*We thank both reviewers for their comments. Responses to specific points raised are as below.*

-Please add a copy of the questionnaire as a supplementary file.

*We apologise that this was not included in the original submission. This omission has now been corrected by inclusion of an additional supplementary file, named ‘S1 Appendix’. We have added reference to S1 Appendix after the description of the questionnaire in the Materials and methods section on line 98.*

-What measures were taken to ensure data confidentiality?

*The information provided by each participant was provided anonymously. Respondents completed a Google Forms questionnaire which did not collect any identifying information. For this reason, it is not possible for respondents to be identified and there are therefore no issues of confidentiality for this study. However, and in line with journal requirements, the anonymous data obtained from the study are shared via a repository through the following site:* [*https://doi.org/10.18746/bmth.data.00000316*](https://eur02.safelinks.protection.outlook.com/?url=https%3A%2F%2Fdoi.org%2F10.18746%2Fbmth.data.00000316&data=05%7C01%7Csbuchan%40bournemouth.ac.uk%7Cbf4344f6fc8e49c4bc8508db9a4d3358%7Cede29655d09742e4bbb5f38d427fbfb8%7C0%7C0%7C638273428578257406%7CUnknown%7CTWFpbGZsb3d8eyJWIjoiMC4wLjAwMDAiLCJQIjoiV2luMzIiLCJBTiI6Ik1haWwiLCJXVCI6Mn0%3D%7C3000%7C%7C%7C&sdata=teFZ8ZBLi8Gs%2FriZCWs3tcwcNIfZUpTlYHGMV889buw%3D&reserved=0)*’.*

-The questionnaire didn’t assess whether participants would personally put pressure on prescribers to get an antibiotic which would be a personal factor. Please add this to the limitations.

*We thank the reviewer for pointing this out and note that this sort of pressuring behaviour by patients has been noted in other studies (we refer to this in lines 66 and also 334). The reviewer is correct however that this is not something we explored in the current study. To acknowledge this, we have added a short section within the discussion (after line 361 of the original manuscript, corresponding to lines 366-372 in the tracked version) to highlight this as a specific limitation of the study.*

-At times the questions seem to be granular asking about some details such as the projected number of deaths due to AR by 2050 to the nearest million (to be specified not as a choice to be selected from several ranges), whereas another question compares the risk of AR to cancer (neglecting some other important leading causes of death worldwide).
-Did the authors compare the risk of AR to other known leading causes of death such as cardiovascular conditions or just cancer? If the answer is no, please comment on the reason why.

*The reviewer is correct that some of our questions required a binary response whereas others were more granular. We elected to keep open-text responses to a minimum in this survey (open text answers only comprised 3 of the substantive questions throughout the questionnaire) to facilitate analysis. With regards the second point about comparison of AR with other leading causes of death, we note that the top 5 leading causes of death in the UK in 2022 were, in order, dementia/Alzheimers disease, ischaemic heart disease, chronic lower respiratory disease, cerebrovascular disease and respiratory malignancy* and so the reviewers query about the omission of a question relating to cardiovascular disease is entirely fair. However, we chose to ask ‘which of the following two options are you more worried about?’. While cancer is not objectively the leading cause of death in the UK, given that ½ individuals will develop cancer in their lifetime and cancer is associated with debilitating treatment regimes, it remains a significant ‘worry’ for the public. It is also a term which is broadly understood by non-healthcare professionals, whereas more specific terms such as cardiovascular disease can be misinterpreted by those unclear as to whether stroke or angina for example are encompassed within the term. We were also mindful to avoid bias for conditions which affect a significantly skewed demographic (e.g. Alzheimer’s disease which preferentially affects women). For these reasons, we selected cancer as one of the comparators. A similar rationale fed into our decision to use diabetes as a comparator. We were also influenced in our decisions here by the Review on Antimicrobial Resistance (citation 11 in our manuscript) which selected ‘cancer’ and ‘diabetes’ as their comparisons for predicted AMR rates in 2050 (page 11 of the report).*

*https://www.ons.gov.uk/peoplepopulationandcommunity/birthsdeathsandmarriages/deaths/articles/deathregistrationsummarystatisticsenglandandwales/2022#:~:text=For%20males%2C%20ischaemic%20heart%20diseases,both%20increases%20compared%20with%202021.

-Line 89: Did the form automatically collect emails of participants?

*Email addresses of participants were not collected by the Google Forms questionnaire. We thank the reviewer for noting that we had not explicitly stated that this was the case however and have added a phrase in line 100-101 to clarify this point.*

-Line 112-113: "Electronic and anonymous agreement was obtained from all participants". How was that done? Were these included in the form or as a separate link?

*Electronic agreement was obtained from all participants as part of the Google Form itself. The beginning of the questionnaire contained a tick box for participants to agree to take part. This was a compulsory requirement and participants could not progress to the questions until this tick box was checked. To add clarity to the Materials and methods section of the manuscript on this point, we have added the word ‘compulsory’ in line 105 (to now read ‘a compulsory checkbox was used to confirm written participant consent’) and have incorporated an additional sentence starting on line 106 to read ‘Participants were therefore unable to continue into the questionnaire without consenting to take part.’.*

-Line 113: Any reason to explain why the response rate was higher in 2020 relative to 2021? Did the authors do more promotion in 2020? Could it be a heightened public consciousness as a result of COVID in 2020?

*The reviewer is correct that in 2020 the questionnaire was open for 2 weeks and garnered 106 responses, whereas the subsequent 2021 opening of the same questionnaire (over 17 days) resulted in only 58 responders. I am however mindful that the first responses were obtained in July whereas the second set of responses were obtained in December, prior to Christmas which may have negatively influenced the number of people responding due to competing commitments. Furthermore, July 2020 in the UK was a time of significant social hesitation (there were still local lockdowns)– it’s possible this may have contributed to people having more time available to complete an online questionnaire. Many of these restrictions had eased by December 2021. The extent of promotion by us was similar at both times but due to the complex social factors at play at the time, I hesitate to draw any conclusions about public consciousness. It’s an interesting wider question however and we thank the reviewer for raising it.*

-Lines 170-176: Does that mean that participants who recognized over-prescription as a contributor to AR development were less likely to recognize the contribution of other factors?

*Compared with those that selected a societal factor (e.g. poor hygiene and sanitation), yes this is correct. The lines referred to here (lines 170-176) are the statistics within the legend to Figure 1. We have tried to convey the biological meaning of these analyses in the text of the manuscript in lines 158-161: ‘Furthermore, individuals selecting contributors not directly related to personal behaviour were more likely to have selected multiple options, indicating greater general knowledge of factors contributing to AR in this cohort.’ However, in response to this comment we have refined the text to try and convey this more clearly. The sentence now reads: ‘Furthermore, individuals that selected ‘over-prescription of antibiotics’ chose fewer additional options compared with those that recognised societal factors, suggesting that individuals that recognise societal factors have a greater general knowledge of contributors to AR (Fig 1)’. This new sentence can be found at lines 160-163 in the tracked version of the manuscript.*

-Lines 201-203: The percentage is not shown in Figure 2 (A).

*We thank the reviewer for highlighting this omission. We have now added the % values to Figure 2A.*

-Lines 382-383: 37% is more like one-third of the participants and not just a few participants.

*We thank the reviewer for pointing out this sentence is misleading. The point that we were trying to make is that fewer participants were aware of societal contributors to antibiotic resistance compared with personal contributors. However, we appreciate that the sentence as written does not convey this clearly. We have therefore amended the sentence improve clarity. The sentence now reads ‘Relatively few participants in our study were aware of societal impacts such as absence of new antibiotic discoveries (37%) or overuse of antibiotics in livestock (50%), compared with >90% that were aware of over-prescription of antibiotics, as contributors to AR.’ This amended sentence can be found at lines 393-396 in the tracked version of the document.*

-Line 421: Is that figure right? Half of individuals will be diagnosed with cancer in their lifetime?

*We thank the reviewer for this question. The figure is correct but on revisiting the sentence we realise that a citation for this specific point was not included and in response new citation 43 has been added (and subsequent citation numbers adjusted accordingly). This statistic comes from Cancer Research UK, with the data ultimately taken from the national cancer registry of the UK. We have however amended this sentence (lines 421-423 in the original manuscript corresponding to lines 433-435 in the tracked version) to better reflect that these data are from the UK rather than worldwide.*
